# Effects of a Specific Pre- and Probiotic Combination and Parent Stock Vaccination on Performance and Bacterial Communities in Broilers Challenged with a Multidrug-Resistant *Escherichia coli*

**DOI:** 10.3390/antibiotics11121703

**Published:** 2022-11-26

**Authors:** Laura Fuhrmann, Jürgen Zentek, Wilfried Vahjen, Ronald Günther, Eva-Maria Saliu

**Affiliations:** 1 Institute of Animal Nutrition, Department of Veterinary Medicine, Freie Universität Berlin, 14195 Berlin, Germany; 2 Fachtierärztliche Praxis für Wirtschaftsgeflügel und Beratung, 39104 Magdeburg, Germany

**Keywords:** *Enterococcus faecium*, FOS, direct fed microbials, microbiota, chicken

## Abstract

Antibiotic resistance poses a risk for human and animal health, leading to a growing demand for effective alternatives. Combining nutritional tools and parent stock vaccination could be an approach to achieve sufficient protection against bacterial infections in poultry. In an *Escherichia coli* O1/O18 challenge trial, we investigated the protective effects of feeding diets containing *Enterococcus faecium* DSM 7134 and fructooligosaccharides (FOS) combined with specific parent stock vaccination in 225 ROSS 308 broilers. Data on performance parameters, intestinal microbial composition and metabolites, and antibiotic resistance genes (*sul1-3*, *dhfr1a*, *SHV-12*) were obtained. *E. faecium* and FOS combined with parent stock vaccination led to the highest body weights, which were significantly higher than those of controls throughout the experiment and decreased the relative abundance of *Proteobacteria* in the crop digesta compared to that in the positive control. However, cumulative feed conversation remained unaffected by the strategies. Birds receiving the pre-/probiotic combination showed lower cecal pH levels and higher crop L-lactate concentrations than the controls, whereas copy numbers of *dhfr1a* (trimethoprim resistance) and *SHV-12* (extended-spectrum beta-lactamase) genes were only decreased in broilers from vaccinated hens compared to those in the challenged control. In conclusion, prophylactic administration of *E. faecium* and FOS in combination with parent stock vaccination can have complementary effects by improving broiler weight gain and stimulating intestinal bacterial metabolism, which may be beneficial for maintaining gut health in terms of *Escherichia coli* infection.

## 1. Introduction

Multidrug-resistant bacteria can pose a major threat to public health [1] and the misuse or overuse of antimicrobials in animal production promotes antimicrobial resistance [2]. Although infections with antibiotic-resistant bacteria in animals often remain clinically undetected, the potential transmission of drug-resistant bacteria to humans or resistance genes to human pathogenic strains may lead to untreatable bacterial diseases [3]. Poultry, in particular, can be considered as one of the main reservoirs of antibiotic-resistant bacteria among livestock. For example, poultry shows the highest prevalence of extended-spectrum beta-lactamase (ESBL)-producing *Enterobacteriaceae* [4].

To address the One Health approach and mitigate the spread of antimicrobial resistance, the European Union (EU) banned antimicrobial growth promoters (AGP) in 2006 [5]. Further restrictions on routine prophylactic and metaphylactic antibiotic treatment of animals were complemented by the EU in 2019, including measures to limit the use of antimicrobials with critical importance for human medicine [6]. However, largely unrestricted antibiotic use in animals occurs in other countries. Thus, the World Organisation for Animal Health (OIE) reported that 42 countries out of 160 (26%) were still using AGP in animal production in 2019, with the highest numbers reported for the Americas (20 out of 35 countries), Asia, the Far East, and Oceania (12 out of 31 countries) [7].

Therefore, given the challenges posed by antimicrobial resistance, there is a growing global demand for safe and effective alternatives to maintain animal health and replace AGP [2]. Research in recent years has indicated that pre- and probiotics can serve as potential substitutes for antimicrobials in poultry nutrition to increase weight gain [8] or reduce gastrointestinal pathogen load [9]. Furthermore, immunization of broiler parent stock has been shown to reduce mortality of progeny associated with different pathogens, such as *Escherichia coli* (*E. coli*) [10], *Ornithobacterium rhinotracheale* [11], or *Clostridium perfringens* [12], as vaccination of hens leads to the transfer of protective antibodies from serum to chicks via the egg yolk [13,14].

Nevertheless, variable outcomes have been reported in different studies. For example, one publication indicated that prebiotic fructooligosaccharides (FOS) significantly improved the feed conversion ratio (FCR) in broiler chickens compared to that in negative controls [15], while another study found no differences for the same parameter in the supplemented group compared to the control group [16]. The probiotic *Enterococcus faecium* (*E. faecium*) NCIMB 10415 significantly increased the amount of lactic acid bacteria in the ileal content and excreta of Ross 308 broiler chickens compared to that of controls [17]. In contrast, another probiotic product containing *E. faecium* showed no effects on total aerobes, *Salmonella* spp., and *Lactobacillus* populations in the small intestine of broilers of the same breed [18]. Furthermore, on the one hand, passive immunization of broilers with egg yolk immunoglobulins (IgY) from broiler breeders (vaccinated with inactivated *E. coli*) appeared to provide effective protection against organ lesions and mortality induced by a homologous *E. coli* challenge [19], but on the other hand, administering hens a polyvalent killed *Enterococcus cecorum* (*E. cecorum*) vaccine failed to protect offspring against homologous or heterologous challenge by *E. cecorum*-specific antibodies in a different experiment [20].

The early implementation of preventive strategies in chicks is of utter importance, as they usually come from hatcheries with high hygienic measures and are challenged by commercial conditions at the farm level. Unlike mammals, which can obtain maternal microbiota during birth, the gastrointestinal environment of newly hatched chicks from hatcheries lacks those maternal bacteria, which may affect immunoglobulin levels [21] and the ability to resist potential pathogens.

Finally, combining multiple early life prevention strategies as opposed to a single measure could be a safe and more effective approach to maintaining poultry health, thus reducing antibiotic use and problems related to antimicrobial resistance development. Hence, with the present study, we aimed to investigate whether a preventive measure combining nutritional tools and parent stock vaccination could serve as a strategy to improve growth performance, promote intestinal health, and reduce the excretion of antibiotic resistance genes in broilers challenged with a multidrug-resistant *E. coli* strain.

## 2. Results

The effects of feeding diets containing *E. faecium* DSM 7134 and FOS combined with specific parent stock vaccination were investigated. Performance parameters, the intestinal microbiota, microbial metabolites, and antibiotic resistance genes (*sul1-3*, *dhfr1a*, *SHV-12*) were evaluated.

### 2.1. Performance

The performances of the different experimental groups are shown in Table 1. Chick mortality occurred only in the first week of life and thus before *E. coli* challenge. Five chicks from the negative control group (Cn), six from the PP (pre-/probiotic combination) group, two from the VAC group (derived from vaccinated hens), and three from the PP-VAC (derived from vaccinated hens and pre-/probiotic combination) group were removed from the pens after death. These animals (16 out of 225; 7.1%) did not show any specific clinical signs beforehand. Chick losses were compensated with reserve animals on the seventh day of life, one day before the challenge, to ensure equal numbers of chicks per pen and feeding group. FCR was corrected for mortality.

Infection with the *E. coli* strain had no negative effects on weight development. However, birds that descended from vaccinated hens (VAC, PP-VAC) showed the highest body weights (BW) throughout the experimental period, which were significantly higher than those of negative (Cn) and/or positive (Cp) controls (*p* ≤ 0.050). The final BW were highest in the PP-VAC group and significantly increased compared to those in the control group and compared those of birds receiving the pre- and probiotic combination without additional parent stock vaccination (*p* < 0.001). For the overall trial period (weeks 1–4), birds that descended from vaccinated hens (VAC, PP-VAC) also showed the highest average body weight gains (BWG), which were significantly higher than those of the negative controls (*p* < 0.001). Additionally, chicks descended from vaccinated hens and receiving the pre- and probiotic combination (PP-VAC) had higher BWG than the positive controls or chicks in the PP group (*p* < 0.001). The same pattern was observed for the cumulative feed intake (FI; weeks 1–4), with the highest values in the PP-VAC group (*p* < 0.001). However, the cumulative feed conversion ratio (FCR) remained unaffected by the different strategies (*p* = 0.449).

Furthermore, the effects on performance were marked during the first two weeks of the trial. Compared to BWG of the positive control group, the BWG of both the VAC and PP-VAC groups increased in the first week (*p* < 0.001), with the PP-VAC group also showing a significantly higher FI than the control or PP groups (*p* = 0.002). In week two, the first week after the *E. coli* challenge, the BWG improved for all treatment groups (PP, VAC, and PP-VAC) compared to those of the control groups (*p* < 0.001). Similarly, an increased FI was observed in these groups compared to that in the negative control (PP, VAC, and PP-VAC) and positive control (VAC, PP-VAC) groups in this week (*p* < 0.001) without any significant effects on the FCR (*p* = 0.253). Interestingly, the FCR was lower in the PP-VAC group during the first two weeks of life than in all other groups, but higher during weeks 3 and 4. Hence, the cumulative FCR was equal for all groups.

### 2.2. Microbial Metabolites and pH in Crop and Cecal Contents

The short-chain fatty acid and lactate concentrations and pH levels in the crop contents and cecal digesta are presented in Table 2.

Overall, lactate was the dominant microbial metabolite in the crop contents of every experimental group. L-lactate concentrations increased most in the groups supplemented with the pre- and probiotic combination (PP, PP-VAC), compared to both control groups (*p* = 0.002). The PP-VAC group also showed a trend toward lower pH values than the control groups (Cn: *p* = 0.054; Cp: *p* = 0.063). Compared to lactate, short-chain fatty acids (SCFA) were present at lower concentrations in the crop digesta, with acetate being numerically most abundant. Moreover, pre- and probiotic supplementation (PP and PP-VAC) significantly decreased the crop propionate concentration compared to that in the negative control (*p* = 0.013), and combination with parent stock vaccination (PP-VAC) resulted in significantly higher i-valerate concentrations than in the positive control (*p* = 0.021).

The lactate concentrations in the cecal contents are not indicated because these values were very low or below the detection limit. Acetate proved to be the most represented SCFA in the cecal digesta of all experimental groups. Parent stock vaccination (VAC; PP-VAC) led to significantly higher concentrations of not only acetate, but also n-butyrate and total SCFA compared those in the negative control (*p* < 0.001). A trend toward higher n-butyrate concentrations was also observed in the PP group compared to that in the Cn group (*p* = 0.058). The highest cecal n-valerate values were detected in the VAC group, which were significantly different from those in the PP group (*p* = 0.021). Similar to the higher concentrations of the mentioned SCFA, the treatment groups (PP; VAC; PP-VAC) showed lower cecal pH values than the negative control group, with birds in the PP and PP-VAC groups additionally showing significantly lower pH than those in the positive control group (*p* < 0.001).

### 2.3. Relative Abundances of Bacteria and Microbial Ecological Indices in Crop and Cecal Contents

**Figure 1:**
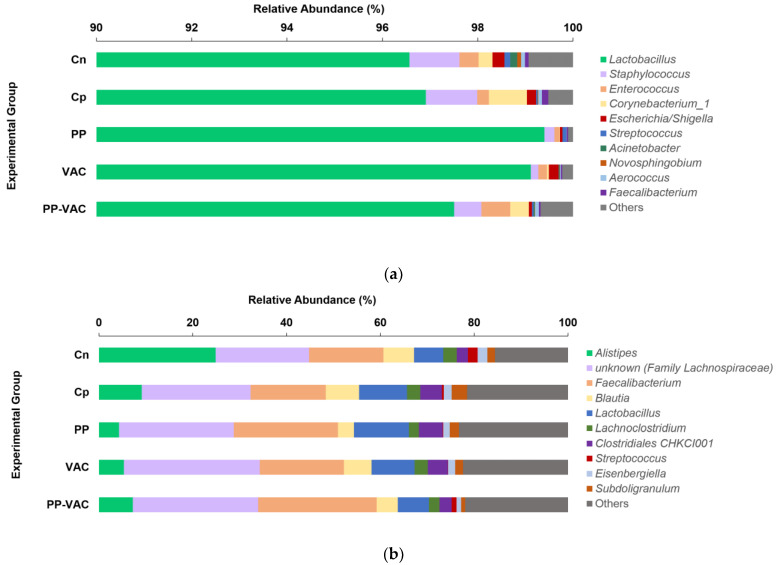
Stacked bar charts presenting the average relative abundances (*n* = 10/group) of main bacterial genera in the (**a**) crop and (**b**) cecal digesta of 28-day-old Ross 308 broilers challenged with *E. coli* O1/O18 on day eight of life. Cn: Negative control, Cp: *E. coli* O1/O18 challenged control, PP: Pre-/probiotic addition, VAC: Parent stock vaccination, PP-VAC: Pre-/probiotic addition and parent stock vaccination.

All data on the ecological indices and relative abundances of bacterial orders, genera, and species are presented in Supplementary Tables S1–S4. The relative abundances of phyla in the crop and cecal digesta are displayed in Table 3, while the relative abundances of the main bacterial genera are shown in Figure 1.

In the crop digesta, microbial richness decreased in all challenged treatment groups (PP, VAC, and PP-VAC) compared to that in the negative control (*p* < 0.001; Table S1). However, microbiota diversity did not differ in the crop digesta of the experimental groups, indicated by Shannon index and evenness. In contrast, Shannon index and evenness increased in the cecal contents of the VAC group compared those of the negative control group (*p* = 0.016 and *p* = 0.012). Microbial richness in the cecal digesta was highest in birds derived from vaccinated hens (VAC, PP-VAC), but only showed a trend compared to that in other experimental groups (*p* = 0.058).

*Firmicutes* was the predominant phylum in the crop contents of the 28-day-old broilers, followed by a clearly lower relative abundance of *Proteobacteria* (Table 3). In contrast, *Actinobacteria* and *Bacteroidetes* were barely detected. The relative abundance of *Firmicutes* increased significantly in the crop digesta of broilers that received the pre- and probiotic combination (PP, PP-VAC) compared to that in the challenged control group (*p* = 0.002) and showed a trend compared to that in the negative control groups (PP: *p* = 0.062; VAC: *p* = 0.082). Only the combination of both preventive measures (PP-VAC) led to a lower abundance of *Proteobacteria* compared to that in both controls (*p* = 0.003). *Bacteroidetes* was only detected in broilers in the Cn and VAC groups, with a significantly higher abundance in the Cn group (*p* < 0.001) compared to that in other group.

In the cecal digesta, *Firmicutes* was again found to be the most abundant phylum, followed by *Bacteroidetes*, and lower abundances were detected for *Proteobacteria*, *Actinobacteria*, and *Tenericutes*. The abundance of *Firmicutes* was higher in the challenged groups than in the negative control group (*p* < 0.001). In contrast, *Bacteroidetes* was relatively less represented in the Cp, PP, VAC, and PP-VAC groups compared to that in the Cn group (*p* < 0.001). As with the crop samples, the PP-VAC group showed a lower abundance of *Proteobacteria* compared to that in the positive control, although this was only observed as a trend (*p* = 0.067).

In the crop contents, an influence of the feed supplements and parent stock vaccination on the relative abundance of bacterial orders was detected. Here, both pre-/probiotic application and parent stock vaccination, alone and in combination, increased the relative abundance of *Lactobacillales* and drastically reduced the abundance of *Rickettsiales* as well as that of *Clostridiales* (Supplementary Table S2). However, significant differences in the relative abundances of *Pseudomonadales* and *Sphingomonadales* were related to the challenge itself, as the abundances of both orders were lower in all challenged groups (*p* = 0.008 and *p* = 0.033).

In the cecal contents, *Clostridiales* was the most abundant order in all experimental groups and significantly more abundant in birds from vaccinated hens (VAC, PP-VAC) compared to that in negative controls (Supplementary Table S2, *p* = 0.001). In contrast, *Bacteroidales* was less abundant in all challenged treatment groups (*p* < 0.001). Furthermore, *Selenomonadales* was not detected in the cecum of the unchallenged control group and in birds fed the pre- and probiotic combination (PP, PP-VAC), but was detected in the VAC and challenged control groups (*p* = 0.018).

As depicted in Figure 1a, the genus *Lactobacillus* dominated the microbiota in the crops of all experimental groups (relative abundance: 96.6%–99.4%, Supplementary Table S3). The VAC group showed the lowest relative abundance of *Streptococcus* in the crop contents, which was significantly lower than that in the Cn group (*p* = 0.016). In crop samples of pre-/probiotic-fed birds (PP, PP-VAC), the relative abundance of *Novosphingobium* was lower than that in the negative controls (*p* = 0.009) and *Clostridium sensu stricto 1* was not detected in these chickens (*p* = 0.032). Moreover, in the PP-VAC group, *Enhydrobacter* and an unknown genus of the Family *Lachnospiraceae* were not found (*p* = 0.019 and *p* = 0.049), but *Facklamia* was detected at low abundance in contrast to all other groups (*p* = 0.073). The control groups contained an unknown genus of the family *Planococcaceae* in the crop digesta, which could not be found in the challenged treatment groups (*p* = 0.004). Furthermore, *Chryseobacterium* was found in non-challenged birds, but not in challenged birds (*p* = 0.001). As the genus *Lactobacillus* showed the highest abundance in the crop digesta, it was further examined at the species level (Supplementary Table S4). Several known *Lactobacillus* species showed numerical differences between experimental groups. However, a trend in statistical differences was observed only for *L. aviaries*, which was not detected in the three treatment groups (PP, VAC, PP-VAC) but was found in low abundance in both control groups (*p* = 0.074).

The following genera were predominant in the cecal contents of all experimental groups: unknown genera of the families *Lachnospiraceae*, *Faecalibacterium,* and *Alistipes* (Figure 1b). *Alistipes* showed the highest abundance in the unchallenged control group (*p* < 0.001). As in the crop contents, the lowest abundance of *Streptococcus* was present in the VAC group, which was significantly lower than that in the Cn group (*p* = 0.012), but at the same time, these birds showed a higher abundance of *Oscillibacter* compared to that in the controls (*p* = 0.014). On the one hand, *Eisenbergiella* and *Lachnospiraceae CAG-56* were significantly less represented in the PP-VAC group than in the negative control group (*p* = 0.008 and *p* = 0.003). On the other hand, *Ruminococcaceae UCG-005*, *Ruminiclostridium 9*, *Ruminiclostridium,* and *Ruminiclostridium 5* were significantly more abundant in these chickens compared to the negative and/or positive controls (*p* ≤ 0.034).

### 2.4. Quantitative Determination of Enterobacterial Antibiotic Resistance Genes and the Corresponding Escherichia coli/Hafnia/Shigella Group

The results of detecting three investigated antibiotic resistance genes (*sul1-3*, *dhfr1a*, and *SHV-12*) and bacterial group *E. coli*/*Hafnia*/*Shigella* in the excreta samples are summarized in Table 4.

Two days after the *E. coli* challenge, increased enterobacterial sulfonamide (sul1-3) resistance was recorded in all challenged groups with a significant higher occurrence of *sul1-3* in the Cp group compared to that in the Cn group. This general trend continued in 14-, 21-, and 28-day old animals. However, trimethoprim (*dhfr1a*) resistance remained unaffected by challenge in the vaccinated groups, in contrast to group PP on days 10 and 21. A similar pattern was observed for the *E. coli*/*Hafnia*/*Shigella* group gene on day ten, which had slightly reduced copy numbers in the vaccinated groups compared to those in the Cp group.

In addition, non-significant differences but numerically lower copy numbers of the extended-spectrum beta-lactamase gene (*SHV-12*) were detected in the vaccinated groups compared to the Cp group on days 14, 21, and 28.

## 3. Discussion

The present study investigated the impact of a preventive strategy including a pre- and probiotic combination and parent stock vaccination on performance parameters, intestinal microbial metabolites, bacterial composition, and excreted resistance genes in broiler chickens.

Regarding growth performance, the final body weights achieved in the negative control group after 28 days of life were comparable to those reported in other studies with Ross 308 broilers [22,23]. Birds originating from vaccinated hens (VAC and PP-VAC groups) showed the highest body weights with significantly improved body weight gains compared to controls in the first two weeks of life. However, feed intake was also increased in these birds, resulting in an unaffected cumulative FCR compared with control birds. Previous studies with different vaccines reported no significant changes in average body weight at 38 days [10] or feed conversion and daily gain [11,24] among broiler flocks descended from vaccinated and control birds, respectively. Nevertheless, the cited publications indicated a lower broiler loss [24] or mortality [10,11] and a higher production index [11,24] in the broiler flocks from vaccinated breeders. The outcomes in the current study might be due to the beneficial effects of parent stock vaccination especially during the early life (first two weeks) of broiler chicks, as maternal IgY antibodies usually disappear between 10 and 20 days after hatching [13]. This could also be the reason for the numerically lower first week mortality of broilers from vaccinated hens (5 out of 90 chicks, 5.56%) compared to that of the immunologically less protected chicks (11 out of 135 chicks, 8.15%) in our study. Nevertheless, as antibodies were not investigated in the present study, these assumptions are only speculative.

However, there may have been synergistic effects with the pre- and probiotic combination. Birds in the PP-VAC group, which were not only from vaccinated hens but also received the additional feed supplements, had the highest body weights throughout the experimental period and highest cumulative body weight gains, which were significantly higher than those of both controls. These results agree with those of previous studies investigating the effects of a commercial synbiotic product containing *E. faecium* (DSM 3530) and FOS in which increased growth performance, villus height/crypt depth ratio, and villus height in the small intestine were reported [25,26]. In addition, another study reported enhanced humoral immune responses against Newcastle disease, infectious bronchitis, and infectious bursal disease after supplementation with the same synbiotic product following vaccination of the chickens [27]. In addition to the two ingredients mentioned above, this commercial synbiotic also contained substances from sea algae, which may have been the reason for the marked performance effects that were not consistently observed in the PP group in our study. Furthermore, we used a multidrug-resistant *E. coli* field isolate, which did not induce negative performance effects compared to the non-challenged control birds. Spatial effects could partly explain this result, as chicks in the negative control group were raised in a different stable than those in the challenged groups in order to avoid cross contamination with the challenge strain. Therefore, it is difficult to conclude whether the applied measures can ameliorate weight losses due to an infection caused by a pathogenic strain. However, directly after challenge with the *E. coli* field isolate, all treatment groups clearly showed better body weight gains than the positive control group during the second week. Overall, the challenge strain obviously exerted only mild effects on the wellbeing of the animals, which makes this multidrug-resistant field isolate a suitable model to study the development and spread of antibiotic resistance in broiler flocks.

L-lactate was the dominant microbial metabolite in the crop while acetate was predominant in the cecal contents of every experimental group, which was consistent with previously reported data [28,29,30,31]. Compared to the negative control group, the presence of the challenge strain in the Cp group appeared to have only a slight, if any, effect on the microbial metabolites, as no significant differences were determined. However, pre- and probiotic supplementation may have had promising effects, since crop L-lactate concentrations increased most in the groups fed the pre- and probiotic combination (PP, PP-VAC) compared levels in both controls groups, and cecal acetate concentrations were higher in the PP-VAC group than in the negative control group. This increase in metabolite concentrations likely led to lower pH values in the crops of the PP-VAC group and cecal digesta of the PP and PP-VAC groups compared to those in the crops of both control groups. A previous in vitro study showed that lactic acid and acetic acid are the main end products in glucose fermentation ofpotential probiotics belonging to lactic acid bacteria [32]. Furthermore, these organic acids can serve as substances with antagonistic properties against potential pathogens, such as *Salmonella typhimurium* or *E. coli,* due to the resulting decrease in pH [32]. The pre- and probiotic combination used in the current study consisted of an *E. faecium* strain and FOS. Other in vitro results indicated the ability of *E. faecium* to metabolize FOS with a rapid pH decline from 6.7 to less than 5.0 within 10 h of incubation at 37 °C [33]. The addition of FOS to the diets probably increased fermentation through the commensal microbiota in the crop and cecum and led to decreased pH values in the respective parts of the intestinal tract. The *E. faecium* strain was likely able to establish well in the crop as a lactic acid bacterium, as it can grow at elevated lactate concentrations. This may have supported the existing microbiota and also account for the significant increase in the relative abundance of *Lactobacillales* in the crop of the pre- and probiotic-fed groups compared to that in controls.

Nevertheless, parent stock vaccination also led to interesting effects in the cecal contents, where levels of acetate, n-butyrate, and thus total SCFA were increased and consequently the pH was reduced compared to the negative control. This could imply that the immune response itself, as a host factor, is sufficient to alter the intestinal bacteria community regardless of diet or feed supplements. As maternal IgY antibodies usually disappear between 10 and 20 days after hatching [13], the observed impact on microbial metabolites in the cecum on day 28 might be due to the long-term effects of derived antibodies in birds from vaccinated parent stock. Maternal IgA for example could be detected in the cytoplasm of goblet cells in chicks [34] and its release on the apical surface of enterocytes may influence intestinal homeostasis and microbiota growth [35,36]. To our knowledge, modulation of microbial metabolites in chickens derived from vaccinated hens has not been described to date. However, several studies have reported effects of vaccination on the cecal microbiota [37,38,39] of chickens. At this point, investigating IgY, IgM, and IgG levels in broiler chickens would likely have further increased the validity of our results.

The higher concentrations of SCFA in the VAC group were accompanied by increased Shannon index and evenness in cecal samples, suggesting that the increased bacterial diversity was related to enhanced production of bacterial metabolites. This is one of the reasons why increased bacterial diversity is often considered to be beneficial. However, this should not be accepted as a general conclusion. An example for the opposite was observed in the crop digesta. Here, lower bacterial richness was detected in all challenged treatment groups (PP, VAC, PP-VAC) compared to the negative control group. This observation correlated with the increased abundance of the genus *Lactobacillus* and decreased pH in the crop digesta, which can be judged as beneficial since it is considered to be a barrier for potentially pathogenic strains. Another study observed no impact of pro- and/or phytobiotic addition to the diet regarding the bacterial richness of the crop contents [28]. However, reduced diversity indices (Shannon index and evenness) were reported in groups supplemented with a *Lactobacillus salivarius* strain and different phytobiotics compared to those in the control group.

Furthermore, a previous study determined no significant effects of a live attenuated IBV (Infectious Bronchitis Virus) vaccine on the Shannon or inverse Simpson indexes of the cecal contents of nine-week-old chickens [37]. Two other publications even reported decreased OTU (operational taxonomic unit) numbers in cecal samples from laying hens vaccinated with a live attenuated *S. enteritidis* vaccine [40] and Cobb broilers vaccinated with a live *E. coli* vaccine [38], respectively. Nevertheless, these outcomes derived from active immunization may not be fully comparable to the impact of maternal immunity on the intestinal microbial diversity indices demonstrated in our study. Similar to our results, ecological indices in cecal contents did not differ significantly between different groups when the diet was supplemented with additional feed ingredients (pro- and/or phytobiotics) [28].

Overall, the relative abundance of the phylum *Firmicutes* was increased in the crops of the PP and PP-VAC groups compared to those in the control groups, which was probably due to better growth conditions for lactic acid bacteria in the FOS-supplemented groups. This increase was at the expense of *Proteobacteria*, which was present in significantly lower abundance in the crops of the PP-VAC group compared to those of the control groups. In addition, the cecal contents of chickens in the PP-VAC group also showed a trend towards lower *Proteobacteria* abundance compared to those in the positive control group. While the phylum *Firmicutes* yields different probiotic strains of the order *Lactobacillales*, the phylum *Proteobacteria* contains genera with potential chicken and human pathogens, such as *Salmonella*, *Escherichia*, *Pasteurella,* or *Campylobacter*. Indeed, the relative abundance of *Firmicutes* in the crop contents of the PP group increased, whereas the abundance of *Pseudomonadales*, an order of the phylum *Proteobacteria* with potentially pernicious bacteria [41], decreased compared to that in the negative control group. Likewise, the PP-VAC group also showed a lower abundance of *Rickettsiales* (*Proteobacteria*) in the crop digesta compared to that in the control groups. As the increased abundance of *Proteobacteria* correlates positively with pro-inflammatory cytokines in chickens [42], a lower abundance of this phylum, but higher levels of *Firmicutes,* may be indicative of a beneficial shift in the microbiota of birds receiving the pre- and probiotic ingredients in combination with parent stock vaccination.

As expected, the genus *Lactobacillus* (phylum *Firmicutes*) dominated the crop digesta of all experimental groups [28,43]. Genera of the phylum *Proteobacteria* (*Acinetobacter* and *Novosphingobium*) and *Clostridium sensu stricto 1* (phylum *Firmicutes*) were not detected in the crop digesta of the PP group and the relative abundance of *Novosphingobium* was also significantly lower in the PP-VAC group compared to that in the negative control group. In a previous study, it was observed that high abundance of *C. sensu stricto 1*, which is very common in the cecum of young chicks [44] and also in other monogastric animals such as pigs [45], was associated with necrotic enteritis in the jejunum [46], indicating potentially deleterious effects in the upper intestinal tract of broiler chickens. *Novosphingobium* spp. are more present in human patients with severe lung disease and can contribute to increased inflammatory responses to smoke exposure in mouse models [47]. Another study reported adverse effects in the liver due to activation of natural killer T cells by *Novosphingobium* in mice [48]. Therefore, bacteria of the genus *Novosphingobium* could also be detrimental to the respiratory or liver function of other animals. Both in the crop and cecal contents, the genus *Streptococcus* (*Firmicutes*) was less present in the VAC group than in the negative control group. Different *Streptococcus* species with pathogenic potential have already been isolated from poultry [49,50,51]. In contrast to *Streptococcus*, the abundance of *Oscillibacter* (phylum *Firmicutes*, *Eubacteriales*) in the cecal contents of the VAC group was significantly higher than that in both control groups. *Oscillibacter* species were positively associated with high feed efficiency in broiler chickens [52] and they express enzymes for the production of butyrate [53], a preferred energy source for enterocytes in the large intestine [54]. Effects at the genus level were also detected in the cecal digesta of the PP-VAC group, where abundances of genera of the class *Clostridia,* such as *Ruminiclostridia* (*Eubacteriales*) or *Ruminococcaceae UCG-005* (*Clostridiales*), were increased compared to those in both control groups and *Eisenbergiella*, a member of the family *Lachnospiraceae* (phylum *Firmicutes, Clostridiales*) was less represented than in the negative control group. *Ruminiclostridium 5* was found at higher abundances in chickens with high abdominal fat percentage and higher BW [55]. In addition, *Ruminococcaceae UCG-005* was highly positively correlated with BW and average daily gain (ADG) in pigs [56]. In contrast, authors of a previous study identified a negative correlation between *Eisenbergiella* abundance, BW, and ADG [57]. However, in the latter study, feed conversion was also negatively correlated with *Eisenbergiella* abundance, suggesting better feed conversion was attributed to this genus [57].

Concerning the excreted antibiotic resistance genes, two days after *E. coli* challenge, increased enterobacterial sulfonamide resistance (*sul1-3*) was recorded in all challenged groups compared to the Cn group, indicating that the strategies employed were not sufficient to reduce excretion of this specific antibiotic resistance gene. This general trend continued in 14-, 21-, and 28-day-old animals. Interestingly, the VAC group, but not the group receiving the pre- and probiotic combination, showed decreased copy numbers of the *dhfr1a* gene. Considered in detail, the lowest copy number levels of the *dhfr1a* gene were observed in the VAC and Cn groups at days 10 and 21, which were significantly lower than those in the PP group. This was accompanied by reduced copy numbers for *E. coli*/*Hafnia*/*Shigella* in the VAC group compared to those in the challenged control group on day 10. As in the VAC and Cn groups, lower *dhfr1a* copy numbers were detected in the PP-VAC group than in the PP group at day 21. These results may be indicative of a potential beneficial effect induced by specific parent stock vaccination against the multidrug-resistant *E. coli* stain, in terms of reduced excretion of *E. coli*/*Hafnia*/*Shigella* and *dhfr1a* genes in the early life of chickens. Indeed, it has already been described that vaccination can be a successful measure to combat antibiotic resistance if it prevents infections and replaces the use of antibiotics [58,59]. Similar to the *dhfr1a* resistance gene, lower copy numbers of the extended-spectrum beta-lactamase gene (*SHV-12*) were detected in the vaccinated groups (VAC, PP-VAC) compared to those in the Cp group on days 14, 21, and 28. However, these differences were not found to be statistically relevant.

Taken as a whole, specific vaccination of parent stocks, and thus improved maternal immunity, can be one approach to achieve protection against antibiotic-resistant bacteria and improve growth performance in the early lives of chickens. When protection by maternal antibodies is depleted in young chickens, it is important to strengthen the immune response. Here, feed supplements were proven to be useful. In a previous study, increased fecal IgA levels were observed in chickens 20 days after vaccination against *Salmonella enteritidis* and administration of probiotic *E. faecium,* compared with a group that received vaccination alone [40]. Additionally, the authors of the cited publication observed a favorable modulation of the intestinal microbiota, including a reduction in the abundance of *Escherichia-Shigella* and increased abundance of *Lactobacillus,* by combining vaccination with probiotic use [40]. These results and the outcomes in our study imply that chickens can benefit from a multimodal preventive strategy that includes both nutritional tools and measures directly aimed at the immune system.

## 4. Materials and Methods

The animal trial was approved by the Regional Office of Health and Social Affairs “Landesamt für Gesundheit und Soziales Berlin” (LAGeSo, G 0029/21).

### 4.1. Broiler Breeders and Vaccination

To investigate the impact of parent stock vaccination and a chosen pre- and probiotic combination on performance, the intestinal microbiota, and their metabolites in broilers, 93 Ross 308 broiler breeders (82 female, 11 male) were purchased at nine weeks of age from a commercial rearing farm in Saxony-Anhalt, Germany. The broiler breeders were kept in five pens and had access to water ad libitum. Feed was offered as meal and presented according to the parent stock production objectives from the breeder [60]. Broiler breeders received grower (week 9–18) and breeder diets (from week 23) based on corn, wheat, soybean meal, and rapeseed meal (Supplementary Table S7). The diets were calculated to meet or exceed the nutrient specifications for growing and adult broiler breeders [61,62]. From weeks 19 to 23, the grower feed was mixed with continuously increasing proportions of the adult breeder feed. The ambient temperature was adjusted at 22 °C. Artificial light was provided for 8 h until 21 weeks of age. From weeks 22 to 26, the duration of lighting increased weekly to a 14 h light and 10 h dark cycle in order to stimulate the development of egg follicles. At 12 and 17 weeks of age, 35 hens were vaccinated intramuscularly with an inactivated autogenous vaccine containing whole-cell *E. coli* O1/O18 field isolate (S20-0172, RIPAC-LABOR GmbH, Potsdam, Germany). The autogenous vaccine was prepared as an oil-based emulsion and used at a dosage of 0.5 mL/hen. Hatching eggs were collected at 32–33 weeks of life and separated according to vaccination or non-vaccination of the parent stocks. Eggs were automatically incubated in a pre-incubator at 37.8 °C and 55% humidity for the first 17 days. From days 17 to 21, incubation took place in the hatcher at 37.3 °C with 58% humidity.

### 4.2. Broiler Chicks and Experimental Groups

After hatching, 225 Ross 308 broiler chicks (male and female, 90 from vaccinated hens, 135 from non-vaccinated hens) were randomly allocated into five experimental groups (Table 5): non-challenged negative control (Cn); *E. coli*-challenged positive control (Cp); diet supplemented with a pre-/probiotic-combination and challenged (PP); descended from vaccinated hens and challenged (VAC); and descended from vaccinated hens, diet supplemented with a pre-/probiotic-combination, and challenged (PP-VAC). The sex of the chickens was determined phenotypically within the first two weeks of life and on day 27. The sex ratios of the experimental groups are shown in Supplementary Table S5.

All broilers were kept in groups with nine chicks per pen and five pens per experimental group in litter-floor pens with Miscanthus shavings (Sieverdingbeck-Agrar, Velen-Ramsdorf, Germany). In addition, 27 reserve birds were kept in three additional pens (separated according to parent stock vaccination and diet) to compensate for possible chick losses. All birds had access to feed and water ad libitum over the total trial period of 28 days. The rooms were pre-warmed prior to animal placement. The initial temperature of 35 °C was kept for 48 h and thereafter gradually reduced by 3 °C per week. After a 3 d period of constant light, a daily cycle comprising 18 h light and 6 h darkness was provided from day four onwards. During the experimental period, the general conditions of the chickens were monitored daily with a focus on: feed and water intake, frequency of respiration, appearance of the plumage, and fecal consistency. Body weight (BW) and feed intake (FI) were recorded weekly to calculate average body weight gain (BWG) and feed conversion ratio (FCR=FI/BWG) with the pen as the experimental unit.

### 4.3. Challenge Strain

At eight days of age, birds in the Cp, PP, VAC, and PP-VAC groups were orally challenged with 600 µL of an *E. coli* field isolate (serotype O1/O18) at a dosage of 3.24 × 10^7^ cfu/mL. The challenge strain was kindly provided by RIPAC-LABOR GmbH (Potsdam, Germany) and showed a multidrug resistance profile against various antibiotics (Supplementary Table S6). To avoid cross-contamination, broilers in group Cn were reared separately from the challenged broilers in another coop.

The *E. coli* challenge suspension was prepared based on a standard protocol [63] with small modifications. Briefly, cryopreserved bacterial colony material was incubated overnight at 37 °C in 10 mL LB broth (LB Lennox, Carl Roth GmbH + Co. KG, Karlsruhe, Germany) with 0.1% glucose (D(+)-glucose, Carl Roth GmbH + Co. KG, Karlsruhe, Germany), two days before the challenge day. The following day, 100 µL of this pre-culture was transferred into 10 mL fresh medium (LB broth with 0.1% glucose) and incubated again at 37 °C for 8 h, before making a 1:2 × 10^7^ dilution in an Erlenmeyer flask. Subsequently, the diluted main culture was incubated again overnight at 37 °C under constant shaking (110 rpm). On the challenge day, the culture was slowly cooled to 23 °C and centrifuged (10,000 × *g* for 20 min at 23 °C). The supernatant was discarded, the cell pellet was resolved in sterile buffered peptone water (Sigma-Aldrich, Taufkirchen, Germany), and the cell density was adjusted to 1.74 × 10^8^ cells/mL. Broilers from the challenged groups received 600 µL suspension orally, applied into the crop. The dispenser tips were changed between pens. Chicks in the Cn group received the same volume of sterile peptone water. To determine the concentration of vital cells, the challenge suspension was serially diluted and plated in duplicate on LB (with 0.1% glucose) agar plates (1.5% Agar No.1, OXOID LIMITED, Basingstoke, England). After incubation at 37 °C for 24 h, an actual concentration of 3.24 × 10^7^ cfu/mL challenge suspension was determined.

### 4.4. Experimental Diets, Prebiotic and Probiotic

Two different experimental diets were offered to the broiler chicks in meal form: a control diet based on soybean meal, corn, and wheat and a diet based on the same ingredients but supplemented with the pre- and probiotic combination. The contents of the diets were calculated to meet the nutrient recommendations as specified by the German Society for Nutritional Physiology for broilers [64]. The feed composition and analyzed nutrient contents of the diets are shown in Table 6 (complete analysis is shown in Supplementary Table S8). The pre-/probiotic combination was selected according to its promising effect on growth inhibition of *E. coli* O1/O18 in a preliminary ex vivo study [65]. A commercial granulated probiotic product containing *E. faecium* DSM 7134 (1 × 10^10^ cfu/g additive, Bonvital^®^, LACTOSAN GmbH & Co. KG, Kampfenberg, Austria) was used at a dose of 0.1 g/kg diet according to the manufacturer’s recommendations for minimum inclusion level. The actual dosage was verified by serial dilution in duplicate on Slanetz and Bartley medium (OXOID LIMITED, Basingstoke, England) agar plates and proven to be 1.01 × 10^9^ cfu/kg complete feed for the starter pre-/probiotic diet and 8.5 × 10^8^/kg complete feed for the grower pre-/probiotic diet. The probiotic strain is authorized in the EU as a zootechnical additive (functional group: gut flora stabilizer) for chicken fattening according to council regulation (EC) No. 1831/2003.

In addition, fructooligosaccharides derived from chicory inulin by partial enzymatic hydrolysis (Orafti^®^OPS, BENEO-Orafti S.A., Tienen, Belgium) served as prebiotic product at a dosage of 10 g/kg experimental diet.

### 4.5. Sampling

At the end of the experimental period, two chickens per pen (ten chickens for each experimental group) were stunned and killed by exsanguination. Crop and cecal contents were filled directly into sterile tubes, the pH was measured, and samples were stored at −80 °C until further analysis of microbial metabolites and intestinal bacteria.

In addition, fresh excreta samples were collected from each pen on days 10, 14, 21, and 28 as pool samples and stored at −80 °C in order to quantify the bacterial *Escherichia*/*Hafnia*/*Shigella* group and resistance genes by qPCR.

During collection of samples and further analysis, protective clothing and sterile gloves were worn to avoid contamination by human handlers. Sampling equipment was renewed or disinfected between animals or pens, respectively.

### 4.6. Analysis of Microbial Metabolites and pH

In crop and cecal digesta samples, concentrations of short-chain fatty acids (SCFA: acetate, propionate, i/n-butyrate, and i/n-valerate) and D- and L-lactate were analyzed as previously described [66]. Briefly, SCFA were examined using gas chromatography (Agilent Technologies 6890N, autosampler G2614A, and injection tower G2613A; Network GC Systems, Böblingen, Germany) with caproic acid as the internal standard. Total branched-chain fatty acids (BCFA) were calculated as the sum of i-butyrate and i-valerate. D- and L-lactate concentrations were measured via HPLC (Agilent 1100; Agilent Technologies, Böblingen, Germany). The pH was measured in the original undiluted digesta samples using an electronic pH meter (Seven Multi pH meter, Mettler-Toledo GmbH, Gießen, Germany) immediately after sacrificing the broilers.

### 4.7. 16S rDNA Sequencing and qPCR

DNA was extracted from 250 mg crop and cecal contents (for 16S rDNA sequencing) or excreta samples (for qPCR), respectively. A commercial extraction kit (QIAamp PowerFecal Pro DNA Kit, Qiagen, Hilden, Germany) was used according to the manufacturer’s protocol, except for an additional lysis step at 65 °C for 10 min, as previously described [65]. Thereafter, the DNA extracts were frozen at −30 °C until further analysis.

The obtained DNA extracts from the crop and cecal contents were sequenced on an Illumina NextSeq500 sequencer (LGC, Berlin, Germany) with two 150 bp-pair reads. After demultiplexing via Illumina bcl2fastq (v. 2.17.1.14), paired reads were combined using BBMerge (v. 34.48). Finally, the resulting 16S rDNA sequences were determined with the QIIME2 pipeline [67] and the SILVA SSU database [68]. Quality control and sequence counts were analyzed using DADA2 [69]. Further details regarding sequence read processing were described previously [28].

Quantification of the bacterial group *Escherichia*/*Hafnia*/*Shigella* and resistance genes *sul1-3* (sulfonamide resistance gene), *dhfr1a* (trimethoprim resistance gene), and *SHV-12* (extended-spectrum beta-lactamase gene) in excreta samples was achieved via qPCR using an AriaMx real-time PCR system (Agilent, Santa Clara, CA, USA) with AriaMx V 1.4 software (Agilent, Santa Clara, CA, USA) and Brilliant II SYBR Green qPCR Master Mix with Low ROX (Agilent, Santa Clara, CA, USA). Primer sequences and annealing temperatures are presented in Table 7. All amplification programs were performed with an initial denaturation step at 95 °C for 10 min, followed by a 30 s annealing time, and an extension at 72 °C for 30 s. Melting curves of the PCR products were inspected in order to review correct amplification of the target gene. Target gene copy numbers/g excreta were calculated from a calibration series with known copy number concentrations.

### 4.8. Statistical Analysis

The results are presented as means with standard deviation or pooled standard error of the mean. Statistical analyses were performed using SPSS 28.0 software (IBM SPSS Statistics 28.0; Armonk, NY, USA). Data were analyzed using ANOVA and post hoc Tukey-HSD for normally distributed data (performance data, pH), and the non-parametric Kruskal–Wallis test with Bonferroni correction followed by the Mann–Whitney test was applied for non-normally distributed data (microbial metabolites, ecological indices of the intestinal microbiota, relative abundance, target gene copy numbers). Differences were considered to be significant at *p* ≤ 0.05 and *p*-values between 0.05 and 0.10 were considered indicative of a trend.

## 5. Conclusions

In the presents study, the beneficial effects (increased BW and BWG, reduced digesta pH, decreased abundance of *Proteobacteria*) obtained by combining both preventive strategies were probably attributed to the better initial conditions of the chicks with parent stock vaccination and favorable modulation of the intestinal microbiota by the applied pre- and probiotic combination. Thus, the combination of parent stock vaccination with pre- and probiotic supplementation provides a promising tool for future strategies to reduce the usage of antibiotics in broilers and consequent development of antibiotic resistance. However, the FCR remained unaffected by the combined strategy and the excretion of antibiotic resistance genes upon challenge with a multidrug-resistant *E. coli* strain was only affected by parent stock vaccination. Further studies are needed to investigate whether maternal immunity together with synbiotics is a sufficient alternative strategy to antibiotics in order to combat severe pathogen challenges in chickens.

## Figures and Tables

**Table 1:** Impact of parent stock vaccination and dietary pre-and probiotic combination on performance parameters ^1^ of growing Ross 308 broilers challenged with *E. coli* O1/O18 on day eight of life.

	Experimental Groups ^2^					
Week	Cn	Cp	PP	VAC	PP-VAC	*p*-Value
Average bodyweight (g)/animal						
0	39.0 ± 0.70	39.8 ± 0.56	39.3 ± 1.04	38.6 ± 0.84	38.7 ± 1.36	0.262
1	92.4 ± 10.9 ^a^	86.6 ± 9.23 ^a^	92.0 ± 6.97 ^a^	104 ± 14.5 ^a,b^	123 ± 13.6 ^b^	<0.001
2	224 ± 27.3 ^a^	223 ± 23.62 ^a^	265 ± 17.0 ^a,b^	277 ± 25.6 ^b^	319 ± 20.6 ^c^	<0.001
3	520 ± 75.3 ^a^	543 ± 52.8 ^a,b^	580 ± 37.1 ^a,b,c^	613 ± 40.5 ^b,c^	680 ± 31.0 ^c^	<0.001
4	929 ± 119 ^a^	985 ± 70.9 ^a,b^	1017 ± 64.3 ^a,b^	1091 ± 31.2 ^b,c^	1144 ± 45.5 ^c^	<0.001
Average bodyweight gain (g)/animal						
1	53.5 ± 10.8 ^a,b^	46.8 ± 8.74 ^a^	52.7 ± 7.78 ^a,b^	65.7 ± 15.1 ^b,c^	83.8 ± 13.7 ^c^	<0.001
2	132 ± 17.1 ^a^	147 ± 14.9 ^a^	169 ± 14.6 ^b^	172 ± 12.9 ^b^	197 ± 9.10 ^c^	<0.001
3	296 ± 48.4 ^a^	310 ± 30.1 ^a,b^	316 ± 21.8 ^a,b^	336 ± 20.2 ^a,b^	361 ± 20.5 ^b^	0.026
4	410 ± 44.7	442 ± 26.7	437 ± 29.7	479 ± 49.5	464 ± 21.0	0.086
1–4	890 ± 119 ^a^	945 ± 70.6 ^a,b^	974 ± 64.4 ^a,b^	1052 ± 31.3 ^b,c^	1106 ± 44.2 ^c^	<0.001
Average feed intake (g)/animal						
1	68.4 ± 12.7 ^a^	71.4 ± 10.9 ^a^	68.7 ± 4.41 ^a^	91.5 ± 18.9 ^a,b^	96.8 ± 14.0 ^b^	0.002
2	203 ± 12.84 ^a^	233 ± 16.2 ^b^	253 ± 6.11 ^b,c^	277 ± 29.5 ^c,d^	287 ± 17.5 ^d^	<0.001
3	368 ± 59.0 ^a^	387 ± 41.4 ^a^	412 ± 34.0 ^a,b^	421 ± 29.0 ^a,b^	477 ± 19.6 ^b^	0.003
4	557 ± 62.0 ^a^	599 ± 41.0 ^a,b^	612 ± 47.2 ^a,b^	633 ± 33.9 ^a,b^	672 ± 33.4 ^b^	0.018
1–4	1197 ± 141 ^a^	1289 ± 88.2 ^a,b^	1347 ± 82.6 ^a,b^	1422 ± 76.2 ^b,c^	1532 ± 64.4 ^c^	<0.001
Feed conversion ratio ^3^						
1	1.39 ± 0.14 ^a,b^	1.54 ± 0.14 ^b^	1.47 ± 0.21 ^b^	1.48 ± 0.16 ^b^	1.17 ± 0.03 ^a^	0.007
2	1.55 ± 0.17	1.60 ± 0.18	1.51 ± 0.12	1.61 ± 0.14	1.47 ± 0.04	0.253
3	1.25 ± 0.02 ^a^	1.25 ± 0.02 ^a^	1.30 ± 0.03 ^b^	1.25 ± 0.04 ^a^	1.32 ± 0.04 ^b^	0.001
4	1.36 ± 0.02 ^a^	1.36 ± 0.04 ^a^	1.40 ± 0.03 ^a,b^	1.33 ± 0.14 ^a^	1.45 ± 0.02 ^b^	<0.001
1–4	1.35 ± 0.04	1.38 ± 0.01	1.39 ± 0.03	1.35 ± 0.07	1.39 ± 0.01	0.449

^1^ Performance data are presented as means ± standard deviation ^2^ Cn: Negative control, Cp: *E. coli* O1/O18 challenged control, PP: Pre-/probiotic addition, VAC: Parent stock vaccination, PP-VAC: Pre-/probiotic addition and parent stock vaccination. ^3^ Corrected for mortality: calculated as total feed intake divided by total gain including weight of deceased birds. ^a,b,c,d^ Different superscripts indicate significant differences within a row (*p* ≤ 0.05). Statistical analyses were conducted using one-way ANOVA followed by post hoc Tukey-test (*n* = 5/group).

**Table 2:** Effects of parent stock vaccination and dietary pre-and probiotic combination on pH and lactate, short-chain fatty acid (SCFA), and branched-chain fatty acid (BCFA) concentrations [μmol/g] ^1^ in crop and cecal digesta of 28-day-old Ross 308 broilers challenged with *E. coli* O1/O18 on day eight of life.

	Experimental Groups ^2^						
	Cn	Cp	PP	VAC	PP-VAC	SEM	*p*-Value
**Crop**							
pH	5.17 *	5.16 *	4.86	4.88	4.74 ^#^	0.052	0.022
L-lactate	20.4 ^a^	22.0 ^a,b,#^	49.6 ^b,c,^*	41.5 ^a,b,c^	52.5 ^c^	3.34	0.002
D-lactate	7.80	12.1	21.8	15.5	34.1	3.07	0.100
acetate	8.31	7.92	10.9	6.47	11.8	0.946	0.396
propionate	0.120 ^b^	0.083 ^a,b^	0.071 ^a^	0.077 ^a,b^	0.070 ^a^	0.005	0.013
i-butyrate	0.087	0.137	0.104	0.101	0.118	0.006	0.176
n-butyrate	0.026	0.025	0.021	0.017	0.016	0.004	0.661
i-valerate	0.010 ^a,b^	0.008 ^a^	0.013 ^a,b^	0.013 ^a,b^	0.019 ^b^	0.001	0.021
n-valerate	0.043	0.033	0.030	0.032	0.024	0.002	0.168
total BCFA	0.097	0.145	0.118	0.114	0.136	0.007	0.181
total SCFA	6.45	8.26	11.1	6.72	12.0	0.896	0.263
**Cecum**							
pH	7.24 ^c^	7.10 ^b,c^	6.75 ^a^	6.88 ^a,b^	6.77 ^a^	0.042	<0.001
acetate	62.3 ^a^	69.6 ^a,b^	81.1 ^a,b^	90.6 ^b^	88.4 ^b^	2.48	<0.001
propionate	8.87	7.82	8.76	10.1	8.95	0.451	0.655
i-butyrate	1.58	1.37	1.22	1.86	1.84	0.092	0.117
n-butyrate	7.80 ^a,#^	12.7 ^a,b^	16.6 ^a,b,^*	20.2 ^b^	19.4 ^b^	1.07	<0.001
i-valerate	1.34	1.41	1.29	1.75	1.72	0.084	0.179
n-valerate	1.24 ^a,b^	1.22 ^a,b^	1.11 ^a^	1.54 ^b^	1.35 ^a,b^	0.059	0.021
total BCFA	2.92	2.78	2.51	3.62	3.56	0.155	0.113
total SCFA	83.3 ^a^	94.1 ^a,b,#^	110 ^a,b^	126 ^b,^*	122 ^b^	3.44	<0.001

^1^ pH values and SCFA and lactate concentrations are indicated as mean with pooled standard error of the mean (SEM). ^2^ Cn: Negative control, Cp: *E. coli* O1/O18 challenged control, PP: Pre-/probiotic addition, VAC: Parent stock vaccination, PP-VAC: Pre-/probiotic addition and parent stock vaccination. ^a,b,c^ Different superscripts indicate significant differences within a row (*p* ≤ 0.05). ^#,^* Different signs indicate a trend towards statistical significance (0.05 < *p* ≤ 0.10) between groups. Statistical analyses were conducted using the Kruskal-Wallis-test followed by Mann-Whitney-U-test and Bonferroni correction for SCFA and lactate concentrations or one-way ANOVA followed by post hoc Tukey-test for pH (*n* = 10/group).

**Table 3:** Impact of parent stock vaccination and dietary pre-and probiotic combination on relative abundance (%) ^1^ of bacterial phyla in crop and cecal digesta of 28-day-old Ross 308 broilers challenged with *E. coli* O1/O18 on day eight of life.

	Experimental Groups ^2^						
	Cn	Cp	PP	VAC	PP-VAC	SEM	*p*-Value
**Crop**							
*Firmicutes*	91.3 ^a,b,#^	91.3 ^a^	97.6 ^b,^*	96.9 ^a,b^	96.7 ^b,^*	0.710	0.002
*Proteobacteria*	8.22 ^b^	7.83 ^b^	2.40 ^a,b^	3.06 ^a,b^	1.92 ^a^	0.711	0.003
*Actinobacteria*	0.202	0.033	0.013	0.052	0.653	0.078	0.152
*Bacteroidetes*	0.059 ^b^	n.d. ^a^	n.d. ^a^	0.003 ^a^	n.d. ^a^	0.006	<0.001
**Cecum**							
*Firmicutes*	73.8 ^a^	88.5 ^b^	92.5 ^b^	92.1 ^b^	91.7 ^b^	1.36	<0.001
*Bacteroidetes*	25.9 ^b,^*	11.1 ^a,b,#^	7.32 ^a^	7.68 ^a^	8.02 ^a^	1.36	<0.001
*Proteobacteria*	0.001 ^#^	0.275 *	0.138	0.202	0.005 ^#^	0.026	0.010
*Actinobacteria*	0.047	0.069	0.047	0.044	0.046	0.006	0.990
*Tenericutes*	0.067	0.006	0.001	0.034	0.012	0.008	0.061

^1^ Data are presented as means with pooled standard error of the mean (SEM). n.d. = not detected. ^2^ Cn: Negative control, Cp: *E. coli* O1/O18 challenged control, PP: Pre-/probiotic addition, VAC: Parent stock vaccination, PP-VAC: Pre-/probiotic addition and parent stock vaccination. ^a,b^ Different superscripts indicate significant differences within a row (*p* ≤ 0.05). ^#,^* Different signs indicate a trend towards statistical significance (0.05 < *p* ≤ 0.10) between groups. Statistical analyses were conducted using the Kruskal-Wallis-test followed by Mann-Whitney-U-test and Bonferroni correction (*n* = 10/group).

**Table 4:** Impact of parent stock vaccination and dietary pre-and probiotic combination on gene copy numbers (log_10_) ^1^ of antibiotic resistance genes and bacterial group *Escherichia coli*/*Hafnia*/*Shigella* in excreta samples of growing Ross 308 broilers challenged with the multi-drug-resistant *E. coli* O1/O18 strain on day eight of life.

		Experimental Groups ^2^						
Target Gene ^3^	Day	Cn	Cp	PP	VAC	PP-VAC	SEM	*p*-Value
***sul1-3***								
	10	4.09 ^a,#^	7.68 ^b^	7.39 ^a,b,^*	6.75 ^a,b^	7.19 ^a,b^	0.330	0.015
	14	6.32 ^#^	7.92	7.33	7.73	8.24 *	0.219	0.053
	21	6.26 ^a^	7.57 ^a,b^	8.35 ^b^	7.60 ^a,b^	6.95 ^a,b^	0.196	0.012
	*28*	7.81	8.55	8.14	8.03	7.72	0.128	0.354
***dhfr1a***								
	10	2.52 ^a^	4.44 ^a,b^	5.65 ^b^	2.37 ^a^	3.33 ^a,b^	0.517	0.010
	14	1.43	4.48	2.37	3.76	3.77	0.523	0.222
	21	2.55 ^a^	5.63 ^a,b^	6.44 ^b^	1.43 ^a^	2.47 ^a^	0.574	<0.001
	*28*	3.38	4.76	2.76	1.43	2.70	0.525	0.080
***SHV-12***								
	10	3.29	2.80	4.58	3.51	4.81	0.457	0.217
	14	3.86	4.08	3.85	1.36	3.08	0.438	0.295
	21	3.63	3.64	3.20	1.36	1.36	0.384	0.124
	*28*	3.99	3.12	2.93	1.36	1.36	0.385	0.097
***E. coli/Hafnia/Shigella***								
	10	8.76 ^a,b,#^	9.51 ^b,^*	9.34 ^a,b^	8.57 ^a^	8.88 ^a,b^	0.135	0.020
	14	8.75	9.05	8.57	8.84	8.81	0.126	0.845
	21	8.87	8.82	9.01	8.82	9.31	0.113	0.396
	*28*	8.84	9.03	9.18	8.75	9.08	0.109	0.703

^1^ Data are presented as means with pooled standard error of the mean (SEM). ^2^ Cn: Negative control, Cp: *E. coli* O1/O18 challenged control, PP: Pre-/probiotic addition, VAC: Parent stock vaccination, PP-VAC: Pre-/probiotic addition and parent stock vaccination. ^3^ *sul1-3*: sulfonamide resistance gene, *dhfr1a*: trimethoprim resistance gene, *SHV-12*: extended-spectrum beta-lactamase gene. ^a,b^ Different superscripts indicate significant differences within a row (*p* ≤ 0.05). ^#,^* Different signs indicate a trend towards statistical significance (0.05 < *p* ≤ 0.10) between groups. Statistical analyses were conducted using the Kruskal-Wallis-test followed by Mann-Whitney-U-test and Bonferroni correction (*n* = 5/group).

**Table 5:** Experimental setup to test preventive strategies (pre-/probiotic combination and parent stock vaccination) in an *Escherichia coli* challenge model with growing Ross 308 broilers.

Experimental Group ^1^	Parent Stock Vaccination ^2^	Pre-/Probiotic Combination ^3^	*E. coli*- Challenge ^4^
Cn	No	No	No
Cp	No	No	Yes
PP	No	Yes	Yes
VAC	Yes	No	Yes
PP-VAC	Yes	Yes	Yes

^1^ Five pens for each experimental group, total number of chicks per group: 45; ^2^ Autogenous vaccine (*Escherichia coli* O1/O18, inactivated, RIPAC-LABOR GmbH, Germany); ^3^ Probiotic: *Enterococcus faecium* DSM 7134 0.1 g/kg complete feed (Bonvital^®^, LACTOSAN GmbH & Co. KG, Kampfenberg, Austria) and prebiotic: fructooligosaccharides 10 g/kg complete feed (Orafti^®^OPS, BENEO-Orafti S.A., Tienen, Belgium) from the first day of life; ^4^ Oral challenge at day eight of life with a multidrug-resistant *Escherichia coli* O1/O18 field isolate (RIPAC-LABOR GmbH, Potsdam, Germany) 3.24 × 10^7^ cfu/mL, 600 µL/chick; birds in Cn group received 600 µL sterile peptone water.

**Table 6:** Composition and analyzed nutrient composition of the experimental diets (as-fed basis). The experimental starter diet was fed on days 1–14 and the grower diet on days 15–28 to Ross 308 broiler chicks.

	Starter		Grower	
	Basal diet	Pre-/Probiotic Diet ^1^	Basal Diet	Pre-/Probiotic Diet ^1^
**Ingredients [%]**				
Soybean Meal	33.55	33.55	30.18	30.18
Maize	32.31	32.31	34.93	34.93
Wheat	24.85	23.85	25.00	24.00
Soybean oil	4.40	4.40	5.48	5.48
Limestone	1.68	1.68	1.54	1.54
Monocalcium phosphate	1.28	1.28	1.08	1.08
Trace mineral and vitamin premix ^2^	1.20	1.20	1.20	1.20
DL-Methionine	0.35	0.35	0.30	0.30
L-Lysine HCL	0.28	0.28	0.22	0.22
Threonine	0.10	0.10	0.07	0.07
FOS ^3^	0.00	1.00	0.00	1.00
**Analyzed Nutrient Composition [g/kg]**				
Dry matter	910	910	914	913
Crude ash	52.9	53.9	49.4	48.7
Crude protein	229	233	213	212
Ether extract	69.9	61.4	73.1	71.4
Crude fiber	28.2	24.9	25.0	27.6
Methionine	7.05	7.62	6.41	5.74
Lysine	13.8	13.5	12.4	10.5
Calcium	8.63	8.94	7.86	7.89
Total phosphorus	6.25	6.44	5.77	5.78
AME_N_^4^ [MJ/kg]	12.6	12.6	12.9	12.9

^1^ Diets additionally supplemented and mixed with the probiotic strain: *Enterococcus faecium* DSM 7134 at an inclusion level of 0.1 g supplement/kg complete feed (Bonvital^®^, 10^10^ cfu/g, LACTOSAN GmbH & Co. KG, Austria). ^2^ Contents per kg experimental diet: Fe (ferrous sulphate), 60.00 mg; Cu (copper sulphate), 12.00 mg; Zn (zinc oxide), 60.00 mg; Mn (manganese oxide), 72.00 mg; I (calcium iodide), 0.54 mg; Se (sodium selenite), 0.42 mg; vit. A, 7200 IU; vit. D3, 1440 IU; vit. E, 96.0 mg; vit. K3, 3.60 mg; vit. B1, 3.00 mg; vit. B2, 3.00 mg; vit. B6, 4.80 mg; vit B12, 24.0 µg; niacinamide, 30.0 mg; folate, 1.20 mg; biotin, 300 μg; D-calcium-pantothenate, 12.00 mg; choline chloride, 960.00 mg. ^3^ Fructooligosaccharides (Orafti^®^OPS, BENEO-Orafti S.A., Tienen, Belgium). ^4^ Nitrogen-corrected apparent metabolizable energy calculated from the chemical composition of the feed ingredients (European Commission Directive 86/174/EEC): 0.1551 × % crude protein + 0.3431 × % ether extract + 0.1669 × % starch + 0.1301 × % total sugar.

**Table 7:** Target gene, sequences, and annealing temperatures of primers used for the detection of resistance genes and bacterial group *Escherichia coli*/ *Hafnia*/*Shigella* in excreta samples.

Target Gene	Primer	Sequence (5′ to 3′)	Annealing Temperature	Reference
*sul1-3*	sul1-3-f	CGATCCGGGGATGGGATTTT	60 °C	[70]
	sul1-3-r	CACCGAGACCAATAGCGGAA		
*dhfr1a*	dhfr1a-f	GGAGTGCCAAAGGTGAACAGC	50 °C	[71]
	dhfr1a-r	GAGGCGAAGTCTTGGGTAAAAAC		
*SHV-12*	SHV-12-f	ATTTGTCGCTTCTTTACTCGC	55 °C	[72]
	SHV-12-r	TTTATGGCGTTACCTTTGACC		
*E. coli*/*Hafnia alvei*/ *Shigella* spp. group	Entero-f	GTTAATACCTTTGCTCATTGA	55 °C	[73]
	Entero-r	ACCAGGGTATCTAATCCTGTT		

## Data Availability

Data supporting the reported results are included in the article and Supplementary Materials.

## Supplementary Materials

The following supporting information can be downloaded at: https://www.mdpi.com/article/10.3390/antibiotics11121703/s1, Table S1: Effects of parent stock vaccination and dietary pre-and probiotic combination on ecological indices of the intestinal microbiota in crop and cecal digesta of 28-day-old Ross 308 broilers challenged with *E. coli* O1/O18 on day eight of life; Table S2: Impact of parent stock vaccination and dietary pre-and probiotic combination on relative abundance (%) of bacterial orders in crop and cecal digesta of 28-day-old Ross 308 broilers challenged with *E. coli* O1/O18 on day eight of life; Table S3: Impact of parent stock vaccination and dietary pre-and probiotic combination on relative abundance (%) of bacterial genera in crop and cecal digesta of 28-day-old Ross 308 broilers challenged with *E. coli* O1/O18 on day eight of life; Table S4: Impact of parent stock vaccination and dietary pre-and probiotic combination on relative abundance (%) of known *Lactobacillus* species in crop digesta of 28-day-old Ross 308 broilers challenged with *E. coli* O1/O18 on day eight of life; Table S5: Sex ratios of broilers in experimental groups, detected phenotypically within the first two weeks of life and on day 27; Table S6: Resistance profile of *E. coli* O1/O18, tested with agar diffusion method. R = resistant, S = sensitive; Table S7: Ingredients and analyzed nutrient composition of the grower and breeder diets, fed to Ross 308 broiler breeders (as-fed basis); Table S8: Analyzed nutrient composition of the experimental diets (as-fed basis), fed to growing Ross 308 broiler chicks. The experimental starter diet was fed on days 1–14 and the grower diet on days 15–28 to Ross 308 broiler chicks.
