# Supplementary material for: Effects of a Specific Pre- and Probiotic Combination and Parent Stock Vaccination on Performance and Bacterial Communities in Broilers Challenged with a Multidrug-Resistant Escherichia coli
Source: Antibiotics (Basel). 2022 Nov 26;11(12):1703. doi: 10.3390/antibiotics11121703 (PMC9774208; doi:10.3390/antibiotics11121703)
Supplement: Supplementary file 1 [file antibiotics-11-01703-s001.zip › antibiotics-2007465-supplementary.pdf]

Supplementary Material

Table S1. Effects of parent stock vaccination and dietary pre-and probiotic combination on ecological indices[1] of the intestinal microbiota in crop and cecal digesta of 28-day-old Ross 308 broilers challenged with *E. coli* O1/O18 on day eight of life.

	**Experimental groups[2]**						
	**Cn**	**Cp**	**PP**	**VAC**	**PP-VAC**	**SEM**	***p*-value**
**Crop**							
Richness	48.2 [b]	36.7 [a,b]	24.9 [a]	27.7 [a]	29.8 [a]	1.78	< 0.001
Shannon index	2.27	2.15	1.84	1.83	2.00	0.053	0.047
Evenness	0.587	0.605	0.575	0.565	0.597	0.012	0.804
**Caecum**							
Richness	161	170	167	181	193	4.05	0.058
Shannon index	3.51 [a]	3.92 [a,b]	3.88 [a,b]	4.03 [b]	3.97 [a,b]	0.049	0.016
Evenness	0.692 [a]	0.763 [b]	0.758 [a,b]	0.777 [b]	0.755 [a,b]	0.008	0.012

[1]Data are represented as means with pooled standard error of the mean (SEM). [2]Cn: Negative control, Cp: *E. coli* O1/O18 challenged control, PP: Pre-/probiotic addition, VAC: Parent stock vaccination, PP-VAC: Pre-/probiotic addition and parent stock vaccination. [a,b] Different superscripts indicate significant differences within a line ($p \leq 0.05$). Statistical analyses were conducted by Kruskal-Wallis-test followed by Mann-Whitney-U-test and Bonferroni Correction (n = 10/ group).

Table S2. Impact of parent stock vaccination and dietary pre-and probiotic combination on relative abundance (%)[1] of bacterial orders in crop and cecal digesta of 28-day-old Ross 308 broilers challenged with *E. coli* O1/O18 on day eight of life.

	**Experimental groups[2]**						
	**Cn**	**Cp**	**PP**	**VAC**	**PP-VAC**	**SEM**	***p*-value**
**Crop**							
Lactobacillales	89.9 [a]	89.8 [a]	97.3 [b]	96.6 [a,b]	96.0 [a,b]	0.808	**0.002**
Rickettsiales	7.56 [b]	7.55 [b]	2.29 [a,b]	2.78 [a,b]	1.78 [a]	0.670	**0.003**
Bacillales	1.08	1.12	0.209	0.173	0.712	0.253	0.671
Enterobacteriales	0.304	0.222	0.109	0.256	0.105	0.067	0.853
Corynebacteriales	0.282	0.801	0.010	0.036	0.399	0.168	0.737
Clostridiales	0.235 [b]	0.310 [b]	0.040 [a]	0.070 [a,b]	0.045 [a]	0.042	**0.030**
Pseudomonadales	0.196 [b]	0.016 [a,b]	0.001 [a]	0.019 [a,b]	0.023 [a,b]	0.021	**0.008**
Micrococcales	0.179	0.085	0.016	0.016	0.250	0.046	0.296
Sphingomonadales	0.136 [b]	0.019 [a,b]	0.001 [a]	0.007 [a,b]	0.033 [a,b]	0.017	**0.033**
Flavobacteriales	0.031 *	n.d. #	n.d. #	0.004	n.d. #	0.004	**0.013**
Betaproteobacteriales	0.015 *	0.008	n.d. #	0.001	n.d. #	0.002	**0.041**
Bacteroidales	0.014	0.002	0.001	n.d.	n.d.	0.002	0.133
**Caecum**							

Clostridiales	63.8 ^{a}	76.2 ^{a,b}	77.9 ^{a,b}	81.9 ^{b}	82.6 ^{b}	1.56	**0.001**
Bacteroidales	25.9 ^{b,*}	11.1 $^{a,b,\#}$	7.32 ^{a}	7.68 ^{a}	8.02 ^{a}	1.36	**< 0.001**
Lactobacillales	8.31	10.6	12.0	9.28	7.74	0.885	0.439
Erysipelotrichales	1.24	1.43	2.33	0.802	1.23	0.219	0.541
Bacillales	0.415	0.086	0.333	0.082	0.136	0.064	0.387
Mollicutes_RF39	0.064	0.136	0.006	0.033	0.131	0.033	0.440
Coriobacteriales	0.044	0.069	0.0468	0.044	0.046	0.006	0.989
Gastranaerophilales	0.014	0.011	0.015	0.013	0.043	0.008	0.952
Enterobacteriales	0.005	0.084	0.055	0.009	0.005	0.015	0.160
Desulfovibrionales	n.d.	0.114	0.049	0.139	0.067	0.024	0.220
Selenomonadales	n.d. $^{\#}$	0.027	n.d. $^{\#}$	0.031 *	n.d. $^{\#}$	0.005	**0.018**

[1]Data are represented as means with pooled standard error of the mean (SEM). n.d. = not detected. [2]Cn: Negative control, Cp: *E. coli* O1/O18 challenged control, PP: Pre-/probiotic addition, VAC: Parent stock vaccination, PP-VAC: Pre-/probiotic addition and parent stock vaccination. [a,b]Different superscripts indicate significant differences within a line ($p \leq 0.05$). [#,*]Different signs indicate a trend towards statistical significance ($0.05 < p \leq 0.10$) between groups. Statistical analyses were conducted by Kruskal-Wallis-test followed by Mann-Whitney-U-test and Bonferroni Correction (n = 10/ group, significant differences are marked in bold, trends in italics).

Table S3. Impact of parent stock vaccination and dietary pre-and probiotic combination on relative abundance (%)[1] of bacterial genera in crop and cecal digesta of 28-day-old Ross 308 broilers challenged with *E. coli* O1/O18 on day eight of life.

	Experimental groups[2]						
	**Cn**	**Cp**	**PP**	**VAC**	**PP-VAC**	**SEM**	***p*-value**
**Crop**							
Lactobacillus	96.6	96.9	99.4	99.1	97.5	0.575	*0.092*
Staphylococcus	1.05	1.05	0.486	0.157	0.574	0.239	0.751
Enterococcus	0.405	0.256	0.292	0.18	0.603	0.107	0.673
Corynebacterium_1	0.290	0.759	0.010	0.037	0.390	0.161	0.631
Escherichia/Shigella	0.253	1.179	0.055	0.204	0.064	0.217	0.524
Streptococcus	0.123 ^{b}	0.030 ^{a,b}	0.082 ^{a,b}	0.007 ^{a}	0.038 ^{a,b}	0.012	**0.016**
Acinetobacter	0.143 ^{b}	0.015 ^{a,b}	n.d. ^{a}	0.019 ^{a,b}	0.023 ^{a,b}	0.016	**0.018**
Novosphingobium	0.084 ^{b}	0.011 ^{a,b}	n.d. ^{a}	0.007 ^{a,b}	0.007 ^{a}	0.009	**0.009**
Aerococcus	0.078	0.074	0.009	0.018	0.078	0.019	0.334
Faecalibacterium	0.076	0.136	0.024	0.026	0.037	0.022	0.373
unknown (Family.Lachnospiraceae)	0.074 *	0.046	0.010	0.015	n.d. $^{\#}$	0.011	**0.049**
Brachybacterium	0.073	0.063	0.002	0.011	0.038	0.019	0.711
Enhydrobacter	0.060 ^{b}	0.006 ^{a,b}	0.001 ^{a,b}	0.001 ^{a,b}	n.d. ^{a}	0.007	**0.019**
unknown (Family.Planococcaceae)	0.053 ^{b}	0.033 ^{a,b}	n.d. ^{a}	n.d. ^{a}	n.d. ^{a}	0.007	**0.004**

Porphyrobacter	0.053	n.d.	0.001	n.d.	0.009	0.007	0.124
Klebsiella	0.047	0.004	n.d.	0.053	0.028	0.013	0.567
Romboutsia	0.043	0.09	0.014	0.018	0.009	0.015	0.310
Enterobacter	0.040	n.d.	n.d.	n.d.	n.d.	0.008	0.380
Microbacterium	0.039	0.007	0.008	0.003	0.189	0.034	*0.098*
Chryseobacterium	0.032 ^{b}	n.d. ^{a}	n.d. ^{a}	n.d. ^{a}	n.d. ^{a}	0.004	**0.001**
Sphingomonas	0.024	0.005	n.d.	n.d.	0.02	0.005	0.152
Kurthia	0.020	0.036	0.003	0.015	0.131	0.02	0.160
Weissella	0.017	0.027	0.006	0.020	0.081	0.009	*0.095*
Yonghaparkia	0.017	n.d.	n.d.	0.002	0.023	0.005	0.682
Agrococcus	0.017	0.002	n.d.	n.d.	0.009	0.003	0.402
Brevibacterium	0.017	n.d.	0.001	n.d.	n.d.	0.003	0.552
Blautia	0.015	0.005	0.001	0.003	n.d.	0.002	0.332
Bacteroides	0.010	n.d.	n.d.	n.d.	n.d.	0.002	0.380
Ruminococcaceae_UCG-014	0.010	n.d.	n.d.	n.d.	n.d.	0.002	*0.073*
Jeotgalicoccus	0.010	0.014	n.d.	0.005	0.02	0.004	0.444
Subdoligranulum	0.007	0.009	0.004	n.d.	n.d.	0.002	0.395
Alistipes	0.006	0.003	0.001	n.d.	n.d.	0.001	0.429
Clostridiales CHKCI001	0.006	0.011	0.001	0.003	n.d.	0.002	0.241
Clostridium_sensu_stricto_1	0.006 ^{b}	0.001 ^{a,b}	n.d. ^{a}	0.002 ^{a,b}	n.d. ^{a}	0.001	**0.032**
Pantoea	0.006	0.058	0.064	0.002	0.014	0.013	0.209
Jeotgalibaca	n.d.	0.003	n.d.	0.001	0.033	0.004	0.125
Facklamia	n.d.	n.d.	n.d.	n.d.	0.017	0.003	*0.073*
**Caecum**							
Alistipes	24.9 ^{b,*}	9.15 $^{a,b,\#}$	4.28 ^{a}	5.32 ^{a}	7.24 ^{a}	1.42	**< 0.001**
unknown *Lachnospiraceae*	19.9	23.2	24.5	28.9	26.7	1.07	*0.073*
Faecalibacterium	15.8	16.0	22.2	18.0	25.3	1.72	0.243
Blautia	6.50	7.12	3.42	5.88	4.49	0.535	0.254
Lactobacillus	6.22	10.2	11.7	9.19	6.64	0.896	0.140
Lachnoclostridium	2.93	2.80	2.14	2.85	2.24	0.113	*0.092*
Clostridiales CHKCI001	2.36	4.64	5.05	4.28	2.60	0.651	0.401
Streptococcus	2.07 ^{b}	0.400 ^{a,b}	0.158 ^{a,b}	0.026 ^{a}	1.08 ^{a,b}	0.240	**0.012**
Eisenbergiella	2.07 ^{b}	1.67 ^{a,b}	1.39 ^{a,b}	1.53 ^{a,b}	0.969 ^{a}	0.105	**0.008**
Subdoligranulum	1.65	3.31	1.96	1.68	0.877	0.254	0.106
Sellimonas	1.19	2.38	1.89	1.91	1.76	0.184	0.417

Christensenellaceae_R-7_group	1.13	0.583	0.552	1.03	0.675	0.098	0.130
Prevotella CAG-873	1.05	1.42	0.827	1.63	0.626	0.166	*0.072*
Ruminococcaceae_UCG-014	0.944	1.56	1.84	1.73	1.52	0.169	0.533
Butyricicoccus	0.906	0.776	1.152	0.984	0.97	0.089	0.521
Shuttleworthia	0.654	1.15	0.793	1.27	0.93	0.084	0.231
Ruminococcaceae_UCG-013	0.614 [b,*]	0.665 [b,*]	0.412 [a,b,#]	0.468 [a,b]	0.364 [a]	0.057	**0.045**
Fusicatenibacter	0.599	1.595	0.386	0.634	0.821	0.156	0.105
Marvinbryantia	0.506	1.79	0.81	1.37	0.575	0.174	*0.052*
Faecalitalea	0.453	0.03	0.04	0.034	0.065	0.054	0.228
unknown Ruminococcaceae	0.448	1.03	0.891	0.979	1.43	0.098	*0.065*
Lachnospiraceae_NK4A136_group	0.444	0.911	0.479	0.58	1.07	0.117	0.155
Erysipelatoclostridium	0.422	0.590	1.59	0.368	0.475	0.195	0.533
unknown Bacillaceae	0.415	0.078	0.329	0.073	0.13	0.064	0.275
Romboutsia	0.409	0.339	0.575	0.586	0.309	0.098	0.701
Ruminococcaceae_UCG-005	0.364 [a]	0.374 [a,b]	0.670 [a,b]	0.839 [a,b]	1.71 [b]	0.175	**0.013**
Ruminiclostridium_9	0.348 [a]	0.318 [a,#]	0.485 [a,b,*]	0.770 [b]	0.851 [b]	0.065	**0.011**
Ruminiclostridium	0.320 [a,b]	0.195 [a,#]	0.571 [b]	0.534 [a,b]	0.618 [*]	0.069	**0.034**
Pygmaiobacter	0.311	0.343	0.326	0.242	0.246	0.037	0.917
unknown Clostridiales_vadinBB60_group	0.311	0.116	0.175	0.185	0.369	0.038	0.193
Tyzzerella	0.306	0.15	0.653	0.257	0.391	0.084	0.297
Lachnospiraceae GCA-900066575	0.285	0.413	0.495	0.567	0.333	0.038	0.409
Clostridia UC5-1-2E3	0.269	0.119	0.14	0.112	0.081	0.02	*0.075*
unknown Erysipelotrichaceae	0.198	0.474	0.176	0.126	0.18	0.041	0.275
Lachnospiraceae CAG-56	0.179 [c]	0.046 [a,b]	0.163 [b,c]	0.021 [a]	0.036 [a,b]	0.019	**0.003**
Flavonifractor	0.178 [#]	0.198	0.428	0.322	0.643 [*]	0.049	**0.021**
Negativibacillus	0.169 [a]	0.239 [a,b]	0.263 [a,b]	0.369 [b,*]	0.194 [a,b, #]	0.022	**0.028**
Merdibacter	0.169	0.313	0.523	0.273	0.513	0.098	0.296
Ruminococcaceae_NK4A214_group	0.155	0.200	0.256	0.366	0.447	0.035	*0.051*
Ruminococcus_1	0.136	0.058	0.094	0.131	0.113	0.014	0.165
Ruminiclostridium_5	0.123 [a]	0.207 [a,b]	0.379 [a,b]	0.549 [b,c]	0.632 [c]	0.057	**0.001**
Clostridiales DTU089	0.119	0.094	0.07	0.219	0.141	0.023	0.646
Ruminococcaceae UBA1819	0.11	0.087	0.103	0.103	0.097	0.016	0.768
Ruminococcaceae_UCG-008	0.107	0.293	0.306	0.055	0.251	0.056	0.263
Ruminococcaceae_UCG-004	0.089	0.101	0.097	0.135	0.118	0.007	0.312
Oscillibacter	0.071 [a]	0.109 [a,b,#]	0.103 [a,b]	0.243 [b,*]	0.150 [a,b]	0.018	**0.014**

unknown Mollicutes_RF39	0.064	0.136	0.006	0.033	0.131	0.033	0.326
Fournierella	0.057	0.086	0.244	0.226	0.041	0.049	0.627
Intestinimonas	0.052	0.038	0.107	0.169	0.297	0.028	*0.083*
Defluviitaleaceae_UCG-011	0.05	0.051	0.084	0.06	0.076	0.008	0.330
Ruminococcus_2	0.05	0.033	0.087	0.035	0.035	0.008	0.189
Intestinibacter	0.048	0.054	0.037	0.075	0.085	0.013	0.862
Lachnospiraceae_UCG-010	0.047	0.051	0.051	0.031	0.059	0.006	0.617
Coriobacteriaceae CHKCI002	0.042	0.059	0.044	0.041	0.041	0.005	0.997
Lachnospiraceae GCA-900066225	0.041	0.039	0.051	0.033	0.074	0.007	0.409
Anaerostipes	0.037	0.076	0.162	0.075	0.22	0.033	0.372
Coprococcus_3	0.036 [a]	0.282 [b]	0.176 [b]	0.146 [a,b]	0.068 [a,b]	0.024	**0.007**
Ruminococcaceae_UCG-009	0.036	0.027	0.011	0.041	0.04	0.005	0.476
Ruminococcaceae_UCG-010	0.035 [a,b]	0.022 [a]	0.062 [a,b]	0.021 [a,b,#]	0.087 [b,*]	0.007	**0.005**
unknown Peptococcaceae	0.030	0.035	0.009	0.044	0.041	0.006	0.241
Caproiciproducens	0.030 [a,b]	0.002 [a,b]	0.017 [a,b]	n.d. [a]	0.084 [b]	0.033	**0.009**
unknown Gastranaerophilales	0.014	0.011	0.015	0.013	0.043	0.008	0.975
Enterococcus	0.013	0.023	0.074	0.051	0.022	0.008	0.167
unknown Clostridiales	0.011	0.004	0.009	0.008	0.021	0.003	0.626
Lachnospiraceae_FCS020_group	0.007 [a]	0.018 [a,b*]	0.032 [a,b]	0.020 [a,b*]	0.074 [b,#]	0.006	**0.003**
Escherichia/Shigella	0.005	0.084	0.055	0.009	0.005	0.015	0.160

[1]Data are represented as means with pooled standard error of the mean (SEM). n.d. = not detected. [2]Cn: Negative control, Cp: *E. coli* O1/O18 challenged control, PP: Pre-/probiotic addition, VAC: Parent stock vaccination, PP-VAC: Pre-/probiotic addition and parent stock vaccination. [a,b,c] Different superscripts indicate significant differences within a line ($p \leq 0.05$). [#,*] Different signs indicate a trend towards statistical significance ($0.05 < p \leq 0.10$) between groups. Statistical analyses were conducted by Kruskal-Wallis-test followed by Mann-Whitney-U-test and Bonferroni Correction (n = 10/ group, significant differences are marked in bold, trends in italics).

Table S4. Impact of parent stock vaccination and dietary pre-and probiotic combination on relative abundance (%)[1] of known *Lactobacillus* species in crop digesta of 28-day-old Ross 308 broilers challenged with *E. coli* O1/O18 on day eight of life.

Experimental groups[2]

	Cn	Cp	PP	VAC	PP-VAC	SEM	*p*-value
L. crispatus	40.9	57.0	37.1	48.9	47.5	2.61	0.159
L. salivarius	19.3	21.3	44.6	35.6	31.5	3.33	0.201
L. reuteri	7.53	4.2	4.43	2.59	5.56	0.666	0.107
L. kitasatonis	11.9	1.49	3.56	2.39	1.15	1.29	0.221
L. vaginalis	2.25	3.01	3.66	4.43	4.34	0.325	0.233
L. pontis	3.61	2.07	1.87	0.889	1.85	0.515	0.574
L. ingluviei	1.37	3.29	0.525	0.173	1.57	0.431	0.387
L. coleohominis	0.778	0.714	0.694	0.864	1.18	0.079	0.726
L. panis	1.14	n.d.	0.571	0.370	0.587	0.184	0.282
L. oris	0.392	0.643	0.171	0.203	0.421	0.070	0.260
L. secaliphilus	0.032	n.d.	0.006	0.007	0.017	0.006	0.341
L. aviarius	0.016	0.034	n.d.	n.d.	n.d.	0.006	0.074

[1]Data are represented as means with pooled standard error of the mean (SEM). n.d. = not detected. [2]Cn: Negative control, Cp: *E. coli* O1/O18 challenged control, PP: Pre-/probiotic addition, VAC: Parent stock vaccination, PP-VAC: Pre-/probiotic addition and parent stock vaccination. Statistical analyses were conducted by Kruskal-Wallis-test followed by Mann-Whitney-U-test and Bonferroni Correction (n = 10/ group).

Table S5. Sex ratios of broilers in experimental groups, detected phenotypically[1] within the first two weeks of life and on day 27 of life.

	**Experimental groups[2]**				
	**Cn**	**Cp**	**PP**	**VAC**	**PP-VAC**
Male (m)	22	24	21	25	23
Female (f)	23	21	24	20	22
Ratio (m/f)	0.96	1.14	0.88	1.25	1.05

[1]Chickens were phenotyped within the first two weeks of age according to the plumage. A control determination was performed on day 27 with attention to the legs, comb formation and coloration. [2]Cn: Negative control, Cp: *E. coli* O1/O18 challenged control, PP: Pre-/probiotic addition, VAC: Parent stock vaccination, PP-VAC: Pre-/probiotic addition and parent stock vaccination.

Table S6. Resistance profile of *E. coli* O1/O18, tested with agar diffusion method. R = resistant, S = sensitive.

**Antibiotics**	**Concentration**	***E. coli* O1/O18**
Amoxicillin	25 µg	R
Ampicillin	25 µg	R
Bacitracin	10 units	R

Cefacetril	30 µg	S
Cefalexin	30 µg	S
Cefotaxime	5 µg	S
Chloramphenicol	30 µg	S
Ciprofloxacin	5 µg	S
Clindamycin	10 µg	R
Doxycyclin	30 µg	S
Erythromycin	30 µg	S
Framycetin	100 µg	S
Fusidinsäure	10 µg	R
Gentamycin	30 µg	S
Kanamycin	30 µg	S
Lincomycin	15 µg	R
Marbofloxacin	5 µg	S
Metronidazol	5 µg	R
Nystatin	100 units	R
Ofloxacin	5 µg	S
Oxacillin	5 µg	R
Piperacillin	75 µg	S
Rifampicin	30 µg	S
Streptomycin	25 µg	R
Sulfamethoxazol	25 µg	R
Sulfamethoxazol/Trimethoprim	25 µg	R
Tetracyclin	30 µg	S
Trimethoprim	5 µg	R
Tylosin	30 µg	R
Vancomycin	5 µg	R

Table S7. Ingredients and analyzed nutrient composition of the grower and breeder diets, fed to Ross 308 broiler breeders (as-fed basis).

	**Grower**	**Breeder**
**Ingredients [%]**		
Corn	32.65	34.70
Wheat	31.00	33.00
Soybean meal	17.00	15.00
Rapeseed meal	9.00	4.00

Soybean oil	4.30	1.46
Limestone	2.00	8.10
Wheat bran	2.00	1.60
Trace mineral and vitamin premix[1]	2.00	1.20
Monocalcium phosphate	0.00	0.81
Vit. D_3 premix[2]	0.00	0.50
DL-Methionine	0.05	0.10
L-Lysine HCL	0.00	0.02
Tryptophan	0.00	0.01
**Analyzed nutrient composition [g/kg]**		
Dry matter	902	923
Crude ash	55.0	83.8
Crude protein	189	146
Crude fibre	35.1	32.0
Ether extract	26.4	37.7
Calcium	8.93	32.5
Phosphorus	3.12	4.67
ME[3] [MJ/kg]	11.9	11.4

[1]Contents per kg diet: Fe (ferrous sulphate), 60.00 mg; Cu (copper sulphate), 12.00 mg; Zn (zinc oxide), 60.00 mg; Mn (manganese oxide), 72.00 mg; I (calcium iodide), 0.54 mg; Se (sodium selenite), 0.42 mg; vit. A, 7200 IU; vit. D_3, 1440 IU; vit. E, 96.0 mg; vit. K_3, 3.60 mg; vit. B_1, 3.00 mg; vit. B_2, 3.00 mg; vit. B_6, 4.80 mg; vit B_{12}, 24.0 μg; niacinamide, 30.0 mg; folate, 1.20 mg; biotin, 300 μg; D-calcium-pantothenate, 12.00 mg; choline chloride, 960.00 mg. [2]Based on corn; vit. D_3 content per kg diet: 1760 IU (Vitamin D3 500, feed grade, ZMC-Europe GmbH, Hamburg, Germany). [3]Metabolizable energy calculated by Hybrimin program (Futter 2008; Hybrimin, Hessisch Oldendorf, Germany).

Table S8. Analyzed nutrient composition of the experimental diets (as-fed basis), fed to growing Ross 308 broiler chicks. The experimental starter diet was fed on days 1 – 14 and the grower diet on days 15 – 28 to Ross 308 broiler chicks.

		Starter		Grower	
**Analyzed nutrient composition [g/kg]**	Unit	Basal diet	Pre-/probiotic diet	Basal diet	Pre-/probiotic diet
Dry matter	g/kg	910	910	914	913
Crude ash		52.9	53.9	49.4	48.7
Crude protein		229	233	213	212
Ether extract		69.9	61.4	73.1	71.4
Crude fibre		28.2	24.9	25.0	27.6
Starch		363	376	391	389
Sugar		46.0	47.0	42.0	53.0
Neutral detergent		113	137	120	134
Acid detergent fibre		40.5	41.0	44.9	42.3
Acid detergent lignin		5.10	4.50	4.70	5.60
Methionine		7.05	7.62	6.41	5.74
Lysine		13.8	13.8	12.4	10.5
Threonine		7.81	7.55	7.22	6.30
Cystine		3.87	3.71	3.56	3.70
Aspartic acid		5.21	5.16	4.68	6.61
Serine		12.4	12.2	11.8	11.0
Glutamic acid		40.9	39.8	38.5	38.8
Glycine		7.59	7.69	7.23	7.03
Alanine		12.3	11.7	11.5	10.5
Valine		19.6	20.4	18.1	16.0
Isoleucine		8.93	9.36	8.13	7.44
Leucine		18.0	18.0	17.4	16.4
Tyrosine		6.73	6.27	5.88	5.96
Phenylalanine		11.1	11.2	10.6	9.52
Histidine		5.27	5.22	4.88	4.52
Arginine		9.27	10.5	10.2	13.4
Proline		14.6	13.9	13.9	11.2
Calcium		8.63	8.94	7.86	7.89
Total phosphorus		6.25	6.44	5.77	5.78
Potassium		8.43	8.80	8.17	8.95
Sodium		1.99	1.78	1.66	1.74
Magnesium		2.61	2.51	2.52	2.38
Copper	mg/kg	16.3	18.9	17.9	19.5
Manganese		91.4	105	95.3	93.4
Zinc		91.3	93.7	89.4	92.8
Iron		247	275	240	232
AME_N[1]	MJ/kg	12.6	12.6	12.9	12.9

[1]Nitrogen-corrected apparent metabolizable energy calculated from the chemical composition of the feed ingredients (European Commision Directive 86/174/EEC): 0.1551 × % crude protein + 0.3431 × % ether extract + 0.1669 × % starch + 0.1301 × % total sugar.
